# IoT-Based Solution for Detecting and Monitoring Upper Crossed Syndrome

**DOI:** 10.3390/s24010135

**Published:** 2023-12-26

**Authors:** Ammar Shaheen, Hisham Kazim, Mazen Eltawil, Raafat Aburukba

**Affiliations:** Department of Computer Science and Engineering, American University of Sharjah, Sharjah P.O. Box 26666, United Arab Emirates; b00084298@aus.edu (A.S.);

**Keywords:** corrective wearables, back brace, hunchback, machine learning, Upper Crossed Syndrome, inertial measurement unit

## Abstract

A sedentary lifestyle has caused adults to spend more than 6 h seated, which has led to inactivity and spinal issues. This context underscores the growing sedentary behavior, exemplified by extended sitting hours among adults and university students. Such inactivity triggers various health problems and spinal disorders, notably Upper Crossed Syndrome (UCS) and its association with thoracic kyphosis, which can cause severe spinal curvature and related complications. Traditional detection involves clinical assessments and corrective exercises; however, this work proposes a multi-layered system for a back brace to detect, monitor, and potentially prevent the main signs of UCS. Building and using a framework that detects and monitors signs of UCS has facilitated patient–doctor interaction, automated the detection process for improved patient–physician coordination, and helped improve patients’ spines over time. The smart wearable brace includes inertial measurement unit (IMU) sensors targeting hunched-back postures. The IMU sensors capture postural readings, which are then used for classification. Multiple classifiers were used where the long short-term memory (LSTM) model had the highest accuracy of 99.3%. Using the classifier helped detect and monitor UCS over time. Integrating the wearable device with a mobile interface enables real-time data visualization and immediate feedback for users to correct and mitigate UCS-related issues.

## 1. Introduction

The move from an active lifestyle to a sedentary one has become prevalent in urban environments, where it is reported that 47% of adults sit between 4 and 6 h daily [1]. Moreover, it has been observed that university students spend over 12 h in sedentary positions [2]. In 2021, there was an average increase of 135 min in inactivity due to jobs becoming more desk-bound and remote [3]. This shift towards remote work and extended periods of sitting has raised concerns over its impact on posture. Prolonged hours spent sitting can lead to the development of poor postures and musculoskeletal disorders. This trend has been visible in the United Arab Emirates (UAE), where inactivity has decreased the level of physical activity by 26.8% [4]. An increase in sedentary lifestyles has led to numerous physical and mental health problems, which increase depression, obesity, and neck/back pain. As the level of inactivity increases, this causes the degeneration of the spine, creating various spinal disorders such as hunchback, forward head posture, and rounded shoulders. These spinal disorders can lead to long term illnesses which affect the back’s posture. Being in a hunched position can lead to the development of Upper Crossed Syndrome (UCS), characterized by an imbalance in the head and shoulder muscles and associated with forward head posture (FHP), hunchback, and rounded shoulders [5]. According to Singla et al. [6], there is a relationship between HP, rounded shoulders, and hunchback, which is known as thoracic kyphosis. Kyphosis is identified when the angle of the spine’s natural curve exceeds 50 degrees [7]. Since there is an association between these different conditions with respect to thoracic kyphosis, this association shows that UCS is related to thoracic kyphosis. When an individual experiences UCS for an extended period, it can lead to hyperkyphosis, which is a severe curvature of the spine, leading to heartburn [8]. Various corrective exercises and wearables can be used to improve posture. The correlation between forward head and hunchback postures with the development of UCS underscores the importance of predicting the likelihood of an individual to experience spinal disorders. Moreover, UCS cannot be detected directly; therefore, clinical features such as rounded upper back, shoulder protraction, and hunchback can be used to identify signs that the individual is experiencing UCS [9]. The lack of an effective, automated method to accurately detect and monitor signs of UCS using wearable technology necessitates the development of an integrated multi-layered framework. This framework aims to improve the diagnosis, monitoring, and treatment of UCS-associated spinal deformities, enabling effective patient–physician interaction and assessing the advantages of corrective wearables. Furthermore, limited studies have utilized a corrective wearable, such as a back brace, to monitor the improvement of the back posture over time.

The detection of UCS and other spinal deformities includes regular checkups with a physician to evaluate the spine. Some preventive measures are taken to reduce UCS’s effect, including corrective exercises such as stretching and chin tuck exercises. These exercises can help strengthen chest and shoulder muscles, which can improve posture. Additionally, a back brace can be worn to correct the curvature of the spine and to reduce the impact of hunchback posture. The development of embedded systems has allowed the use of wearables for numerous applications, namely in tracking and monitoring an individual’s health. Therefore, a wearable device can be used to diagnose and detect various posture problems and monitor the progression of a disorder over time [10,11]. One such approach involves the use of inertial measurement units (IMUs), which can identify how forward the head is and how effective the wearable device is in mitigating the disorder [10,11]. Therefore, it is essential to retrofit a conventional back brace which can observe the vital signs of UCS and monitor posture problems. This framework can identify spinal disorders that are correlated to UCS and monitor an individual’s posture overtime. This paper expands on the current literature on classifying posture problems using wearable devices with the following contributions:

- This paper proposes a framework that enables the integration of a back brace and enables the interaction between a patient and a doctor for treatment progression.
- The framework automates the detection and monitoring process by observing vital signs of UCS and using machine learning techniques to facilitate and coordinate effective interactions between patients and physicians.
- The framework tracks the correction of the spine over time and the effectiveness of using the back brace to improve posture deformities.

The rest of this paper is organized as follows: A literature review of postural problems and their various implementations are presented in Section 2, the methodology and its details are presented in Section 3, the implementation of the framework and its details are discussed in Section 4, the results of the different classification models and the framework are presented in Section 5, and the impact of the project and its future implications are discussed in Section 6.

## 2. Related Work

Posture problems have become prominent in society, and various systems have been developed to detect and monitor these posture problems. This includes using wearables that can detect and monitor poor sitting and posture habits. The commercialization and incorporation of wearable devices in everyday life have made it possible to identify posture problems and correct their effects. A study by Simpson et al. [12] investigated the importance of real-time postural detection and monitoring systems in improving an individual’s back posture. Several systems were developed to identify these postural disorders by employing various sensor configurations. Table 1 presents a list of wearables that were used to assess a person’s posture.

Table 1 shows that most papers in the literature that deal with spinal detection use an inertial measurement unit (IMU) that contains an accelerometer, gyroscope, and magnetometer, yielding all three axis planes. Since IMU sensors can detect spinal disorders, they can be applied to different wearable devices [13,14,15,16,17,18,20,21,22,23,24]. Specifically, some papers dealt with musculoskeletal disorders that are related to UCS such as identifying hunched and slouched back [14], tracking and monitoring the sitting posture [15], and embedding IMU sensors in clothing to continuously monitor back posture [17,19,20]. These studies show that it is possible to form a unified system that can detect, track, and monitor signs of UCS. Overall, this review indicates how a smart wearable device can be utilized to help diagnose and treat UCS by examining the postures that are correlated with it and monitoring brace wear.

Since UCS is an imbalance of the head and shoulder muscles, one of the clinical features that indicate UCS is FHP. FHP is caused by maintaining an abnormal posture for an extended period, especially while sitting. During that period, individuals often assume a posture characterized by the head’s protraction and spine flexion [26]. Therefore, three approaches have been devised to classify forward head posture: capturing how forward the head is, measuring the curvature of the spine, and measuring muscle stiffness levels [10,25,27]. According to Lee et al. [25], a vision-based detection system was formed using a commercial depth camera. The detection system uses an algorithm that computes the distance between the head and the torso and then compares it to the threshold distance. Consequently, this helps determine how forward the head is, and based on the result, the system then delivers haptic warnings using a neckband. As the head exceeds the threshold, the neckband sends subtle warnings that indicate that the head is too forward, making the user correct their posture. The system achieved an accuracy hit rate of 98% with a false alarm rate of 2%. This accuracy rate shows that implementing a vision-based system can be used to detect FHP; however, this setup only applies when the user is seated and in front of a camera, which is not always the case.

Another system, developed by Wu et al. [27], comprises a wearable posture monitoring system. This system utilizes a combination of a three-axis gyroscope, a three-axis accelerometer, and a magnetometer to evaluate poor postures by measuring the angle of the spine. This occurs by placing two sensors at the back and head occipital bone, which helps determine the upper body angle. As the head goes forward, the angle increases and could exceed the threshold, alerting the user. Meanwhile, a third approach to tackling FHP was developed by Rohal et al. [9], which profiles muscle stiffness levels via vibration signals using a back brace. An array of accelerometers was used to capture and characterize motor vibrations at different distances to profile muscle stiffness. As the head becomes too forward, the signal travels a longer distance and is later captured. This would indicate FHP and be a sign that the head is not aligned properly with the spine. The identification of FHP can be used as a sign of UCS. Furthermore, FHP can lead to an increased curvature of the upper back and contribute to the development of hunchback.

Aside from wearables, some systems can be utilized to potentially detect UCS. According to Silva et al. [28], a system was developed to determine and differentiate subtypes of aphasia that occur due to a stroke. This classification method utilizes acoustic frequencies from pathological speech production and a comprehension analysis to distinguish various aphasia types. This existing framework could potentially be adapted to identify signs of UCS. As UCS is linked to postural imbalances, it may exhibit correlations with motor speech disorders or specific neuromuscular patterns. These neuromuscular imbalances can be associated with UCS. Stress can be another correlation to UCS with the detection of stress in patients. Nair et al. [29] studied the effects of different posture positions on stress. The results showed that participants with an uptight posture had higher pulse pressures and were less likely to be stressed out. On the other hand, slumped participants were more likely to be stressed out. Another study by Hackford et al. [30] analyzed stress by comparing different posture positions. Using pressure sensors, it was found that patients who are less hunched have lower blood pressure rates and are thus less stressed. A classification model was formed by Hong [31] to distinguish between different types of stress, such as emotional and physical stress. Blood oxygen saturation signals can be correlated to the different types of stress using a deep learning-based model. The unique physiological response signals for each stress type can then be used to diagnose chronic stress-induced musculoskeletal imbalances, including UCS. Overall, these observations show how stress can be an indicative factor for UCS. However, one of the main obstacles of these setups is finding a correlation between stress and musculoskeletal disorders directly. Therefore, aside from neuromuscular patterns and stress disorders, hunchback and slouched postures can be a major sign of UCS. Various systems that classify hunchback posture can serve as potential indicators correlated with UCS.

Current research aims to detect specific posture deformities, such as hunchback and slouched postures, in individuals. These postural deformities can serve as vital indicators that an individual may be experiencing UCS. Moreover, a hunchback posture is identified as a significant contributor to UCS. Hunchback, also known as kyphosis, is an excessive curvature of the thoracic spine that appears to be rounded and is caused by poor posture, which leads to a muscular imbalance. Various systems were developed to detect hunchback posture using wearables, as viewed in Table 2.

Each of these systems utilizes an IMU sensor to classify hunchback postures. Jiang et al. [32] demonstrated that a vest design can be used to detect hunchback posture. A Self-Powered Sitting Position Monitoring Vest (SPMV) was developed by attaching IMU sensors at various locations in the vest. The sensors generate electrical signals, which are processed using a random forest classifier. The algorithm yielded an accuracy of 96.6% and was able to recognize precise sitting postures such as hunchback. A study by Fathi et al. [14] detected and classified hunchback and slouched postures. Detection occurs using e-textile and inertial sensors, including an accelerometer, gyroscope, and magnetometer, to detect each axis (x,y,z). The inertial sensors were placed in three different locations in a patient’s back using resistive stretchable fabric. The data were then collected by having the patient form incorrect postures, and then a filter was applied to generate graphs from each sensor and axis. A classification model was used to show the possibility of the posture being hunchback or slouched, which had an accuracy rate of 85%. Even though the study focuses on Ankylosing spondylitis, which is a form of spinal arthritis, the inertial sensors can still be applied to detect hunchback, which is a sign of UCS.

Other configurations include identifying postural deformities, which also encompasses hunchback and slouched postures [33,34]. A study by Gupta et al. [33] used classical machine learning algorithms such as isolation forest and support vector machines (SVM) to classify normal and abnormal postures. The system used three accelerometers situated at various locations in the spine. Then, by using these classification models, it was able to attain an accuracy rate of up to 99.3%. Meanwhile, Farnan et al. [34] used a shirt with built-in magnets that work with a magnetic sensor placed above the body’s sternum. Alongside classifying hunched and slouched postures, the system was also able to identify postures in different directions (left, right, forward, and backward). Determining posture directions can improve prior system configurations. These various observations show how different approaches are used to classify specific postures, which can help identify signs of UCS and monitor its prevalence within the general population over time.

## 3. Methodology

**Figure 1:**
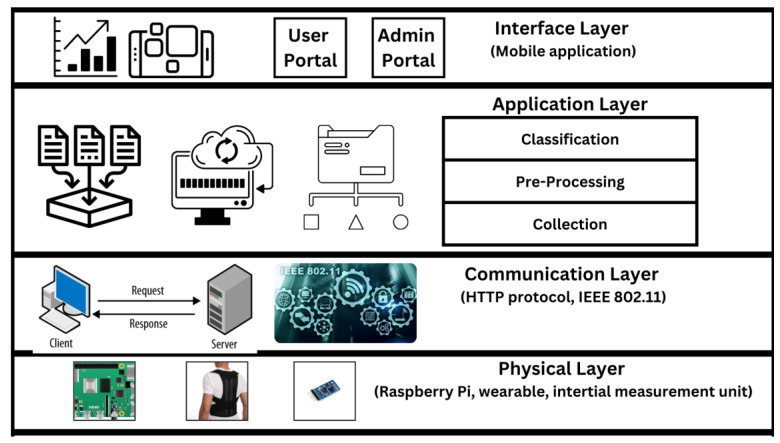
Smart brace proposed framework.

A system in the form of a wearable device can be utilized to detect and monitor improper postures. According to the literature, IMU sensors can be attached to the wearable device to collect posture data. Afterwards, the posture data are processed using a classification algorithm, and the classification result will be displayed to the user to track their posture variation overtime. The relationship between the different components in detecting and monitoring improper posture is shown in Figure 1. The figure shows how the different layers are integrated in the framework. The framework includes the physical layer, communication layer, application layer, and interface layer. The physical layer simulates the connection between the hardware devices to the communication layer, such as the IMU, microcontroller, and other sensors. The communication layer is a link from the physical to the application layer. This layer ensures that the hardware components transmit the necessary readings to the application layer for pre-processing and classification. The transmitted readings are collected, pre-processed, and classified in the application layer, which detects the possibility of improper posture. Finally, the classified readings are transmitted to the interface layer to allow different users to interact and observe the classified posture over time. This framework shows the system’s interactions and functionalities, allowing the detection and monitoring of improper posture.

### 3.1. Physical Layer

This layer is the foundation for the entire system and contains multiple components that are then interfaced with the other layers. The physical layer is the lowest layer, which is associated with the connections between the physical devices. The physical connection is between the wearable, the microcontroller, and the other sensors, such as the IMU. These sensors are interfaced with one another to transmit readings to the application layer by connecting the IMU sensors to a multiplexer, establishing a connection to the microcontroller, and pre-processing and classifying the readings to detect improper posture. However, to establish a link between the physical connections and the posture classification, there should be a transmission link that interfaces the physical and the application layers. This link will help transmit and classify the posture readings.

### 3.2. Communication Layer

Above the physical layer, there is the transmission of data to the server. This transmission occurs through the communication layer, facilitating communication between physical components and the application layer. This layer employs diverse network protocols like HTTP, 5G, IEEE 802.11, and NFC [36]. The communication layer serves as a channel for the data collected from the physical layer, forwarding them to an internal server within the application layer. This transmission enables subsequent data processing and classification. Upon collection, a microcontroller initiates bulk data transfer to the server, triggering a request. Within the server infrastructure, the received data are stored and processed according to classification algorithms, including machine learning and deep learning models. The resulting classifications show the user’s posture and are stored within the server’s data. This classification process allows for the continuous observation and analysis of posture changes over time. Subsequently, the classified data undergo transmission to a mobile interface. This interface interacts with the server, facilitating user engagement by requesting and displaying the classified postures. Users can interact with the interface to view their posture classifications and track changes overtime.

### 3.3. Application Layer

After establishing a link between the physical layer and the server, the readings are transmitted to the server, and then they are pre-processed and classified in the application layer. The role of the application layer is to extract the relevant posture readings and to classify the improper postures that are related to UCS. The application layer includes three components, which ultimately lead to the detection of UCS. These components include data collection, pre-processing, and classification. Data collection occurs after the server and the physical layer form a link that transmit the readings. These readings are then pre-processed into readable and useable information, which is then used in classification via the different classification models. These classification models can either be time-dependent or time-independent and include various machine learning and deep learning models. The result of the classification will be interfaced with the user in the form of a mobile interface, displaying the outcome of the classified readings.

#### 3.3.1. Data Collection and Pre-Processing

To detect hunchback posture from IMU data, an AI-based classification model can be developed. Classification can be time-independent or dependent, with either approach yielding the classified posture readings. The data have the noise filtered out and outliers removed, with the data being transformed into a suitable range. A common way to transform the data is scaling, where the features are scaled, and there are different ways to scale data, including normalization, standardization, and discretization. These different scaling techniques are used when dealing with features of different scales; therefore, scaling the features will make the values uniform, making classification tasks easier. Different features from the dataset are analyzed, and the dimensionality of the data is reduced to remove unnecessary features. Having pre-processed the data, a classification model can be trained to classify the readings.

In the second approach, a different set of data was employed. A previously conducted study by Fathi et al. [14] provided accelerometer and gyroscope data that were specifically collected in relation to hunchback posture. This dataset offered valuable insights into the intricacies of the posture, as it was generated by analyzing patterns and trends in the collected data. In contrast to the first approach, where time was not considered, the second approach emphasized the significance of sequence in the data. The order in which the measurements are taken is a crucial factor in understanding the dynamics of a person’s posture and the development of a classification model.

#### 3.3.2. Data Classification

By collecting and pre-processing data, a classification model can be built to classify the posture readings and to detect any abnormalities. It is important for the classification model to recognize patterns in the data rather than relying solely on the exact data values. This is because there can be slight variations with different user readings. These variations consider changes in the environment, including body shape, movement patterns, and proximity. By assuming that time is independent to classification, various machine learning algorithms can be explored for classification purposes. On the other hand, considering the element of time requires using more sophisticated deep learning methods such as long short-term memory (LSTM). LSTM is effective in handling sequential and time-based data. Capturing long-term temporal dependencies is beneficial in the field of bioinformatics and the detection of health problems using wearable technology [37]. Evaluating different models can be beneficial in understanding the performance of each classification model and the advantages that certain models have over others.

The evaluation of the models occurs by assessing various metrics for each model, such as classification accuracy, recall, precision, and F1 score. To compute these results, a confusion matrix is formed to display the classification result. The matrix has two classes: an actual class and a predicted class. These two classes have four cases, which are true positive (TP), true negative (TN), false positive (FP), and false negative (FN). In the case of TP and TN, these refer to the ability of the model to correctly predict and match the outcome as positive and negative classifications, respectively. Inversely, the FP and FN cases occur when the model falsely predicts a positive and negative classification, respectively. These cases help define the various classification metrics which are used to assess the performance of a model. The classification accuracy shows the ratio of correctly predicted cases out of all of the different cases. Meanwhile, the recall or true positive rate is the ability of the model to correctly identify positive instances out of all positive instances, while precision is a measure of how correctly the model predicted positive results among the different instances it predicted as positive. To incorporate recall and precision together, the F1 score provides a balance between recall and precision by taking their harmonic mean. Equations (1)–(4) quantify each of these four metrics as mathematical formulas [38]. Other metrics such as the area under curve (AUC) examines the area under the receiver operating characteristic (ROC) curve and represents the ability of the model to distinguish between the classes. Mathematically, the AUC is the integral of the ROC as a function of the false negative rate (FPR), and it can be described using Equation (5). Finally, the test loss is a penalty applied to an incorrect prediction and indicates how bad a model is in predicting a single example. Utilizing the built-in libraries in python version 3.11.2 helps compute these metrics to identify and assess a model’s performance.

(1) $$\mathrm{Accuracy}=\frac{\mathrm{TP}+\mathrm{TN}}{\mathrm{TP}+\mathrm{TN}+\mathrm{FP}+\mathrm{FN}}$$

(2) $$\mathrm{Recall}=\frac{\mathrm{TP}}{\mathrm{TP}+\mathrm{FN}}$$

(3) $$\mathrm{Precision}=\frac{\mathrm{TP}}{\mathrm{TP}+\mathrm{FP}}$$

(4) $$F1\;\mathrm{score}=\frac{2\;\times \mathrm{Precision}\;\times \;\mathrm{Recall}}{\mathrm{Precision}+\mathrm{Recall}}$$

(5) AUC=∫ROC d(FPR)

### 3.4. Interface Layer

After establishing a link between the physical and application layers, the result of the classification in the application layer will be displayed to the user in the interface layer. In this layer, there are two different types of users: an admin and a regular user. An admin deals with managing accounts and records, dealing with the different posture readings, adding or removing a wearable, and deleting different accounts from the interface. On the other hand, a regular user can register an account, log in, log out, start detection, associate and dissociate the wearable from the user, delete the account, and display their own postural data on the interface. Having additional user types, such as a physician, can allow an interaction to occur between the user and the physician. This additional feature can help track the user’s posture over time and observe the improvement in the posture. As different users interface with the application, this interface can help build a link between the user and the physical hardware. This link is established by transmitting, pre-processing, and classifying the readings, which are sent through a transmission link to the mobile interface. Creating an interface layer allows different users to view and interpret the results obtained in the application layer. Therefore, establishing a link between the different layers is crucial to observe posture over time.

## 4. Implementation

**Figure 2:**
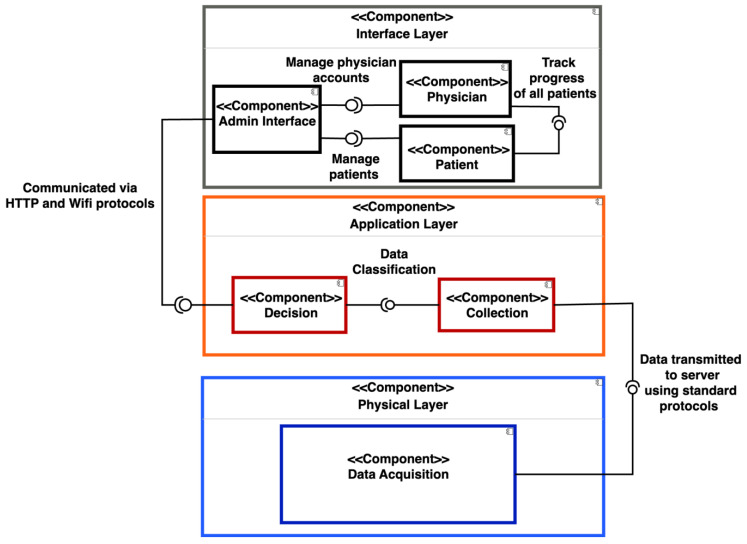
Component diagram of the different layers.

To implement and validate the framework, a component diagram in Figure 2 shows how each layer’s different components interconnect to form a smart back brace that detects and monitors UCS. The Raspberry Pi, IMU, and other hardware components are connected in the back brace to form the data acquisition component at the physical layer. This unit acquires posture readings from the user, which are used for classification. A communication protocol such as HTTP and WIFI are used to transmit the posture readings from the data acquisition unit to the application layer for pre-processing and classification. As a classification decision is formed, the result will be communicated to the interface component using the standard protocols. The interface shows the classified results for the users to view and track. The interface component also has an admin that can manage users. The users include the physician and the patient, where the physician can view and track the data of all of the patients. This component diagram helps build the framework in which the processes of detection and monitoring occur. The framework functions as an Internet of Things (IoT) device, emphasizing the crucial need for seamless communication between the different users. The Raspberry Pi collects data and transmits them to the local server. Subsequently, AI models classify the gathered information into posture readings. Through a mobile app, both the user and their doctor can monitor the user’s back posture progress by accessing the stored data. This functionality enables the doctor to evaluate the effectiveness of the prescribed back brace treatment. Additionally, individuals without a diagnosis or medical consultation can utilize the app to identify signs of UCS. The different components require some sort of communication protocol that can only be delivered using an IoT device. Therefore, an IoT device helps to form an integrated, efficient, and automated solution that collects and classifies results in real time.

### 4.1. Physical Layer

**Figure 3:**
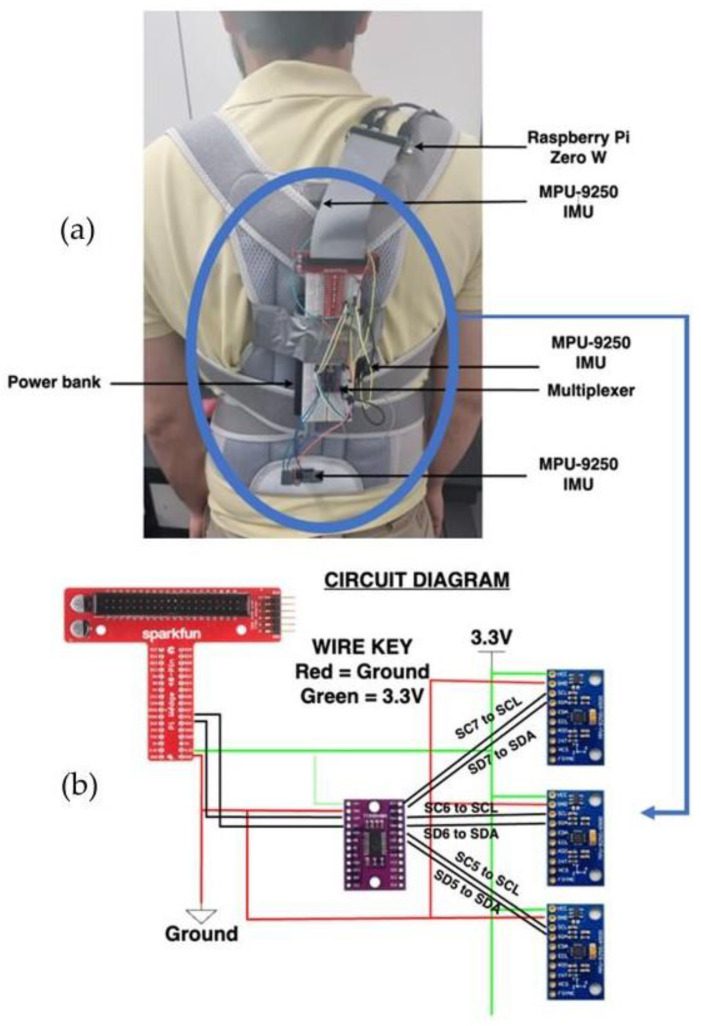
Hardware connections. (**a**) Physical implementation and (**b**) circuit diagram for the hardware components.

Implementing the physical layer connects the three IMU sensors to three distinct locations to collect and transmit posture readings from the gyroscope and accelerometer. These IMU sensors use a multiplexer, which is then connected to a wedge, leading to the Raspberry Pi. The Raspberry Pi is powered through a 10,000 mAh power bank for mobility and power consumption purposes. Forming the connection between the IMU sensors and the Raspberry Pi, Figure 3a shows a top-level view of the different hardware connections on the back brace. Figure 3b shows the circuit diagram of the hardware components, where each IMU sensor is interfaced with a multiplexer. Using the multiplexer gives each IMU a separate address, allowing them to be used simultaneously during detection. The IMU sensors are affixed to the back brace alongside the breadboard and the Raspberry Pi; however, it is important to note that these sensors lack flexibility and are not designed to bend or adapt to changes in the device’s curvature. Therefore, the bending radius of the device is considered to be very small, i.e., negligible. Each of these hardware components plays a role in posture classifications as follows:

1. Raspberry Pi Zero W: The chosen model for the Raspberry Pi is the Zero W model, which is a compact model that is lighter than other microcontrollers and has 40 GPIO connectors. The processor clocks at 1 GHz with 512 MB of ram. Meanwhile, it has a micro-SD slot where the OS is stored and a mini-HDMI port. By utilizing the Raspberry Pi’s compact size and ports, the IMU can be connected to process readings. The Raspberry Pi is from the Raspberry Pi Foundation (Cambridge, England).
2. MPU-9250 Inertial Measurement Unit: This is a three-axis inertial measurement unit (IMU) that contains an accelerometer, gyroscope, and magnetometer. These sensors will be used to detect the motion of the person, which is then used for posture classification. The IMU module is from UIOTEC (Shenzhen, China).
3. TCA9548A Multiplexer: An I2C multiplexer is used, as three IMU sensors from the same manufacturer cannot be interfaced with the Raspberry Pi, as the manufacturer assigns all sensors with the same address of 0 × 68. The multiplexer allows multiple connections, and it provides eight I2C channels as input and connects directly to the Pi’s I2C bus.
4. Power Bank: A 10,000 mAh power bank is used to power up the Raspberry Pi and is also attached to the brace to continuously power it as it is used throughout the day. The power bank is from Promate technologies (Taipei, Taiwan).
5. Back Brace: The brace serves as a posture corrector and provides back lumbar support. The back brace is used to place the hardware components on, and they are mounted on certain positions of the brace.

To interface the hardware components with each other, the following procedure should be taken. Figure 3b shows a circuit diagram, depicting the connections between the sensors. All three IMU sensors are linked to the multiplexer via two connections. Each sensor is individually attached to one of the channels of the multiplexer, utilizing the Serial Data (SDA) and Serial Clock (SCL) lines. The multiplexer, in turn, is linked to the Raspberry Pi’s SDA and SCL pins on the I2C bus, facilitating communication between devices. Notably, both the sensors and the multiplexer draw power from the 3.3 V pin on the Raspberry Pi, ensuring their operational functionality. Using the IMU sensors to collect posture data and transmit them to the server, this allows the posture readings to be used for pre-processing and classification. However, to allow the data to be transmitted to the server for classification, a communication link must be established between the physical and application layers.

### 4.2. Communication Layer

After the physical layer, the data are transmitted to the server via the communication layer. This layer enables the transmission and interaction between the IMU sensors and the Raspberry Pi with the internal server located at the application layer. Various communication standards are utilized such as HTTP and WiFi to transmit data. To transmit the IMU readings for classification, they are sent through the Raspberry Pi server. An HTTP request is executed for the Raspberry Pi server, where the data are sent in bulk, which includes the readings taken from every second in that interval. A pc server is set up to obtain the data from the Raspberry Pi, storing them in the server. In the PC server, a machine learning classification model is applied to the transmitted data, with the classified data being stored in the server. This will then determine the possibility of a user experiencing UCS and monitor their posture over time. After transmitting the data to the classifier, the result is displayed on the mobile application. The mobile interface will make an HTTP request to the server to register and log in, and after sending the request to the server, it will display the classified posture.

### 4.3. Application Layer

As a link is formed between the physical and application layers, the readings are transmitted to the server, which are then pre-processed and classified. In total, 18 features are obtained from the accelerometer and gyroscope sensors from the three axes. We failed to see any variations from the gyroscope sensors; thus, their respective features were dropped, reducing the dimensionality of the data. By reducing the dimensionality of the data, six features were used for classification. In classification, two approaches were implemented. The first approach was based on time dependence and included various ML and neural network (NN) classifiers. These classifiers include K-nearest neighbors (KNN), Naïve Bayes, decision trees, random forests, Multi-Layer Perceptron (MLP), and SVM. These classifiers are well suited for analyzing tabulated types of data, as they can effectively process and classify the tabular IMU data without considering the temporal aspect of the data. Each classifier has its own underlying principles and methodologies to classify the data based on the patterns and the relationships present within the features and the labels.

**Figure 4:**
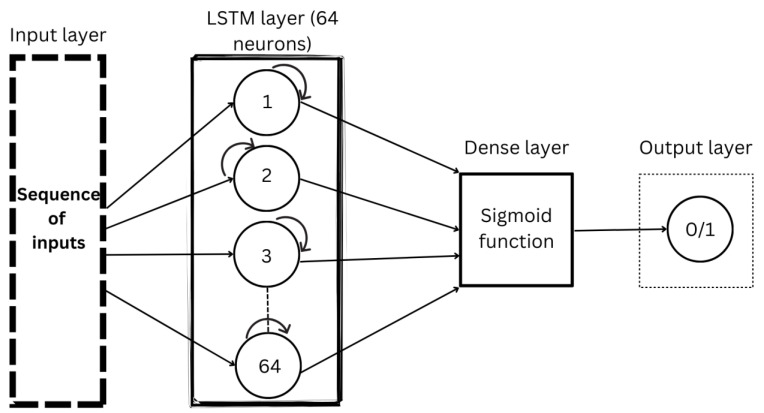
LSTM model architecture.

On the other hand, an LSTM model was implemented to deal with time-dependent data. It is well suited for analyzing IMU data with a temporal aspect. The LSTM architecture that was implemented consists of multiple layers, with the input shape defined based on the dimensions of the IMU data, which are the six IMU measurements. This model consists of two layers used to classify the posture, as seen in Figure 4. The first layer is an LSTM layer with 64 neurons added as the first layer. This layer processes the sequential data, capturing long-term dependencies and retaining the memory of past information. Subsequently, a dense layer with a sigmoid activation function is added to produce the final classification output. The sigmoid function, σ(x), is quantified in Equation (6), where it takes the input value, x, and outputs a value between 0 and 1. Since the output ranges between 0 and 1, it acts as a probability estimate, making the sigmoid function a suitable activation function in binary classification.

(6) $$\sigma \left(x\right)=\frac{1}{{1+e}^{-x}}$$

### 4.4. Interface Layer

**Figure 5:**
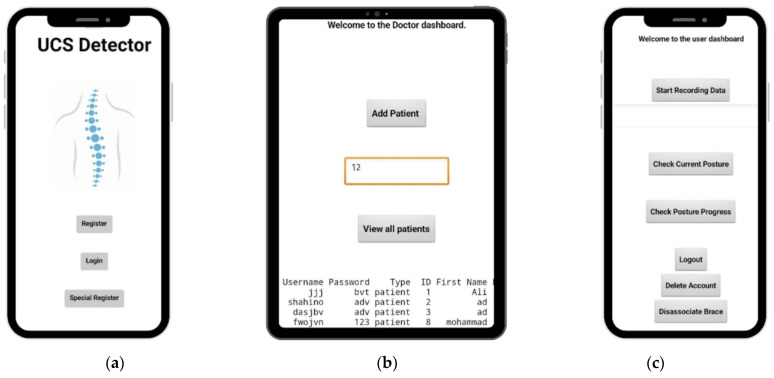
UCS mobile application: (**a**) log in and registration page, (**b**) doctor dashboard, (**c**) user dashboard.

Classifying the posture readings based on the aforementioned techniques requires the use of an interface to interact with the user by developing a mobile application. The mobile application allows different individuals to register and log in, as Figure 5a shows. Different individuals, such as physicians and patients, can use the mobile application. For example, in Figure 5b, the physician interacts with the application to access a patient’s data and see how their posture has improved over time. This builds a framework between the physician and the patient to monitor the patient’s posture and the effectiveness of the current treatment. Meanwhile, the patient can associate the brace by scanning a QR code to start recording data and check their current posture, as displayed in Figure 5c.

**Figure 6:**
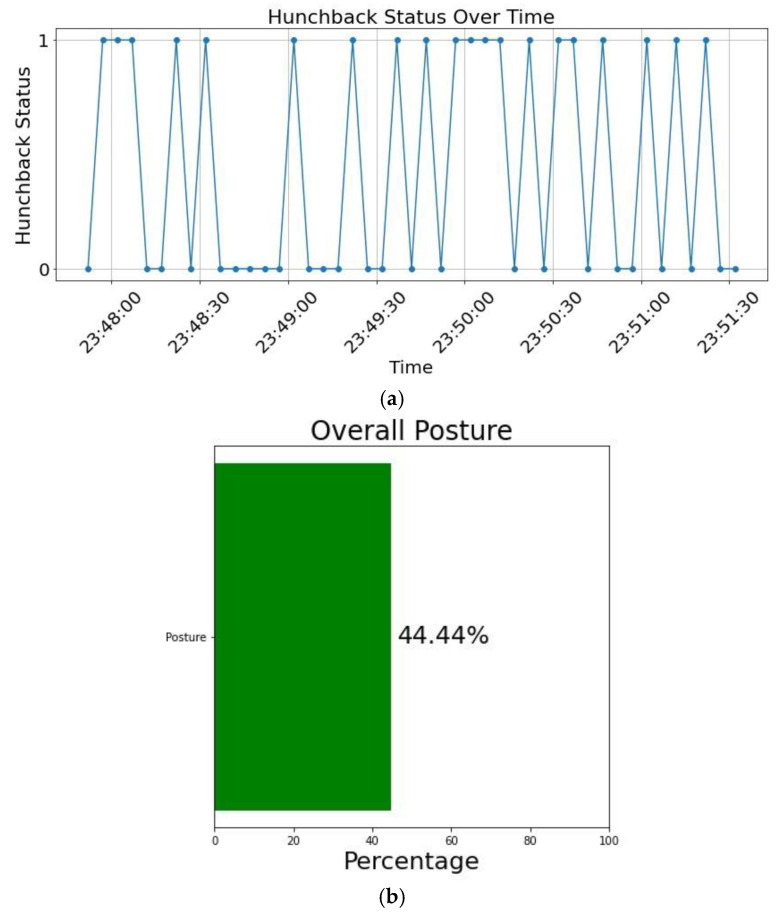
Hunchback status: (**a**) frequency of hunchback and (**b**) overall posture.

The patient can check their posture progress over time and see how often they were in a hunchback position during that period. If the user is in a hunchback position for more than 50% of the time, then this can be a sign which shows that the user is experiencing UCS and should consult their doctor. For clarity, Figure 6a,b display their respective graphs. Figure 6a illustrates the user’s hunchback status over time, showcasing two cases: hunchback and non-hunchback. This duality is depicted in the figure by assigning a value of 1 to denote a hunchback status and 0 for a non-hunchback status. These values enable binary classifications that are observable over time. As the user frequently maintains a hunched position for extended periods, this raises the possibility of the user experiencing signs of UCS. Figure 6b shows that if the user maintains a hunched position for less than 50% of the time, then there are no observable signs of the user suffering from UCS. Otherwise, it might be a strong indication that the user is exhibiting symptoms of UCS. The frequency curve in Figure 6a affects the percentage of hunchback during the entire duration, as evident in Figure 6b. If the frequency of hunchback increases and exceeds 50%, then the bar graph will shift to the right and become red. On the other hand, if the frequency of hunchback decreases and is less than 50%, then the bar graph will shift to the left and become green. The feature of graphing the posture over time helps the physician assess whether the current back brace is effective in improving the posture and if there are signs of improvement. Different users interface with the mobile application, allowing the doctor to monitor a patient’s posture and observe the effectiveness of the current treatment plan. This reduces the number of patient visits to the clinic, reducing hospital fees. Furthermore, storing the history and details of the patient’s posture in a server can help the doctor plan future treatment plans.

## 5. Testing and Results

### 5.1. Experiment Setup

The dataset used for classification is in the form of tabulated, continuous, and numerical values. All data were derived from three different three-axis IMU sensors placed on the brace along the back of the cervical, thoracic, and lumbar spine. These placements were in accordance with the sensor placements seen in the study by Fathi et al. [14]. In the study by Fathi et al., the testing was carried out on five subjects who were asked to perform incorrect postures associated with AS. Thus, for the data collection for this dataset, five subjects were used to collect data in a similar manner. All subjects were asked to wear the brace and perform their daily activities in a healthy, straight posture. In the second phase of data collection, they were asked to perform a hunchback posture while recreating the same activities performed in the first phase. This allowed for variation in the data, as the subjects had their data recorded while being idle (sitting, standing) and while walking. The initial data recordings accounted for all accelerometer and gyroscope data in all three axes, totaling 18 different features. Upon data derivation, the cleaning process began by examining all features to determine the best features in terms of variation. Only six features affected classification in the end, which were the accelerometer data from the three sensors. The other 12 features, notably all gyroscope features, were not used to train the model as they did not contribute to the model, and that would assist in reducing the dimensionality of the dataset. Furthermore, the dataset displayed several outliers due to common sensor errors, which were removed to ensure that outliers do not have negative effects on the classification model. In the end, the features chosen for the dataset were Sensor 1 accelerometer z axis (Az1), Sensor 2 accelerometer x and z axes (Ax2 and Az2), and finally, all accelerometer axes for Sensor 3 (Ax3, Ay3, and Az3).

The configuration of this system is made up of two main hubs: the brace and the server machine. The brace is powered by a Raspberry Pi Zero W2, which is interfaced with the three IMU sensors to collect data from the user and send it to the server for classification. Meanwhile, the server handles all data storage, classification, and functions. To power this, a Windows desktop machine is used as the local server. Communication between the server and the brace is carried out through local Wi-Fi. Both hubs are set up as Flask servers that communicate with each other using HTTP requests. On the server, multiple routes are created to account for all functions needed by the system to classify the posture of a user. Additionally, the mobile application allows the user to connect to and interact with the brace by utilizing a similar communication model with HTTP requests. This setup makes it possible to test and evaluate the efficacy of the back brace against different models to verify the possibility of detecting and monitoring UCS.

### 5.2. Model Testing

After pre-processing and cleaning the data, the dataset was tested on time-independent and time-dependent approaches based on multiple classifiers. These classifiers were tested to determine the one with the best accuracy on the dataset. These classifiers were selected since they are often used for classification and prediction. All classifiers in this section were run through a hyperparameter tuning using the GridSearchCV library to determine which set of hyperparameters performed the best on the dataset. The evaluation was conducted on a testing set, yielding various evaluation metrics for each classifier. These metrics include accuracy, AUC score, macro-average precision, recall, and the F1 score.

While exploring different methods, a rule-based approach was taken into consideration to use data from the IMU to classify hunchback posture. Thresholds were identified by visualizing histograms for each feature to determine potential breakpoints that could separate the two classes. The histograms revealed that the accelerometer values for the two classes overlapped, making it challenging to define a set of rules that could accurately distinguish between the two postures. Additionally, the accelerometer values were found to be highly sensitive to the specific context in which they were collected, which further complicated the development of a rule-based classification approach. The IMU data were slightly different from the previous run at some testing runs. The patterns in the data remained. However, the values differentiated from the previous data. This would sometimes render the thresholds less effective and require modification to fit the new deviation in the data. Therefore, classical ML and neural network models were used to classify the posture readings into hunchback and non-hunchback positions to detect and monitor the possibility of the user experiencing UCS over time.

#### 5.2.1. Decision Trees

**Figure 7:**
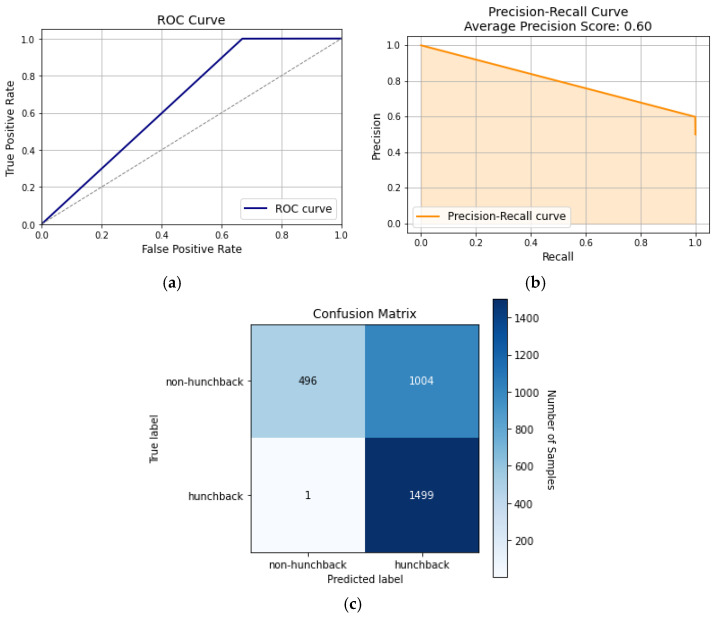
Decision tree curves: (**a**) ROC curve, (**b**) precision–recall curve, and (**c**) confusion matrix.

This approach forms a tree-like model and recursively divides the dataset into subsets. Nodes are created and applied to the entire dataset until it reaches the leaf node, which determines the algorithm’s output. According to Figure 7, the decision tree model is not the most effective model for posture classifications due to the complex and variable nature of the dataset, which differs depending on the user; therefore, a dynamic model should be used instead.

Figure 7 shows the ROC curve, precision–recall curve, and confusion matrix for the decision tree model. Figure 7a shows that the AUC is 62%, which indicates that the model was not able to distinguish between hunchback and non-hunchback positions in most cases. Meanwhile, the precision–recall curve in Figure 7b displays the relationship between the model’s precision and recall, which had an average precision score of 60%, whilst the macro-average precision and recall scores were 80% and 67%, respectively. This variation demonstrates that a decision tree favors precision over recall, meaning it is ineffective in determining all positive cases. Finally, the confusion matrix in Figure 7c shows how correctly (or incorrectly) the model was able to distinguish hunchback positions against non-hunchback positions. Even though there is a high percentage of TP and TN scores, there remains a proportionally high percentage of FP cases at 33.5%. This can result in issues where the smart brace falsely attributes hunchback and UCS to the patient, making the patient follow unnecessary treatment plans which have no effect.

#### 5.2.2. Naïve Bayes

**Figure 8:**
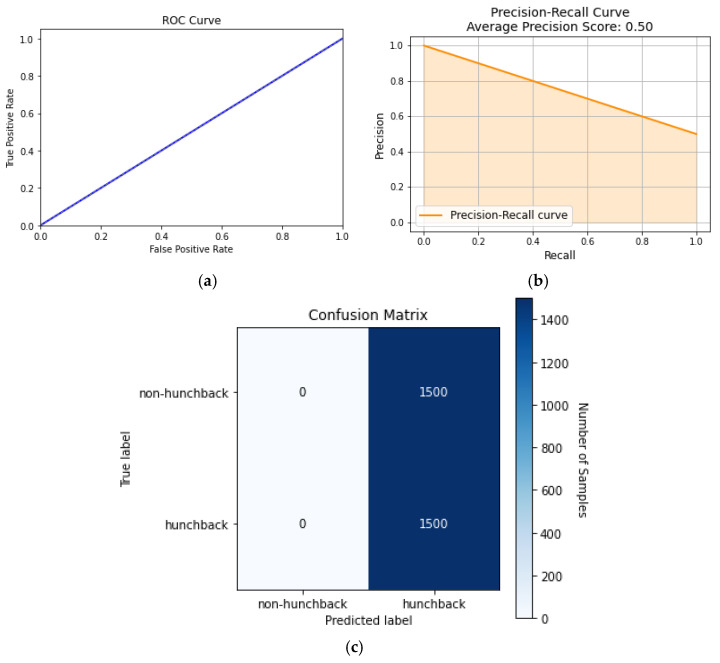
Naïve Bayes curves: (**a**) ROC curve, (**b**) precision–recall curve, and (**c**) confusion matrix.

Naïve Bayes is an ML model that is based on Bayes’ theorem of probability, which describes the probability of an event based on prior knowledge of conditions that might be related to the event. By assuming that the features are conditionally independent, the probability of each feature and class is calculated, and then it makes predictions on unlabeled data. Although Naïve Bayes has document and text analysis applications, it falls short on medical classification. This shortcoming can be seen in Figure 8, where Naïve Bayes performs poorly compared to other algorithms.

Since the Naïve Bayes algorithm assumes that the features are independent, it will perform predictions based on that assumption. However, the different features of the dataset are based on the spine, which are dependent on each other. This makes Naïve Bayes a poor algorithm to use for posture classification. This can be seen in Figure 8a, where the AUC is 50%, which means the model cannot distinguish between hunchback and non-hunchback positions. Figure 8b shows how the precision–recall curve has a score of 0.50, which means that the model was able to correctly predict the posture 50% of the time. Meanwhile, the confusion matrix in Figure 8c indicates that the TP and FP rates are each 50%, indicating that the model randomly predicts if the posture is hunchback or not.

#### 5.2.3. KNN

KNN performed the best out of all the time-independent classifiers since it is robust to noise as it classifies based on the labels of the K-nearest neighbors, and noise would not influence the majority class of the K neighbors. Determining distinct decision boundaries becomes a hard task when the data intersect, as seen from the previous classifiers. KNN operates by categorizing a fresh example according to its K-nearest neighbors’ class labels. Overlapping data indicates that there may be neighboring instances belonging to different classes, which KNN can leverage by recognizing the nearest neighbors of a new instance, irrespective of their class.

**Figure 9:**
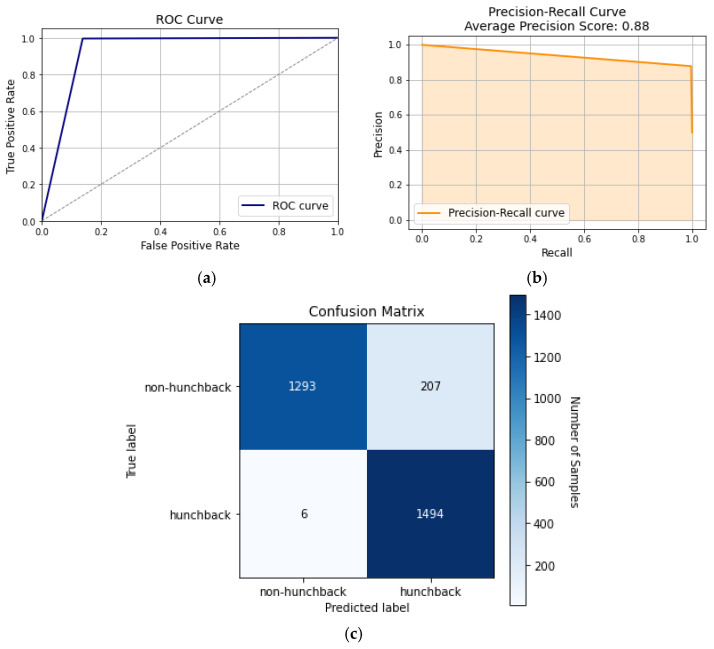
KNN curves: (**a**) ROC curve, (**b**) precision–recall curve, and (**c**) confusion matrix.

Figure 9 displays the performance of the KNN model and how it has an AUC of 93%, as seen in Figure 9a. Figure 9b is the precision–recall curve, which shows that it has lower false positive and negative rates, significantly lower than Naïve Bayes or decision trees. The confusion matrix in Figure 9c shows that the KNN model was able to predict the truth 2787 times in a total of 3000. Meanwhile, false labels rarely occur at 6 and 207 times, respectively, showing how well it can distinguish between hunchback and non-hunchback positions.

#### 5.2.4. Random Forest

**Figure 10:**
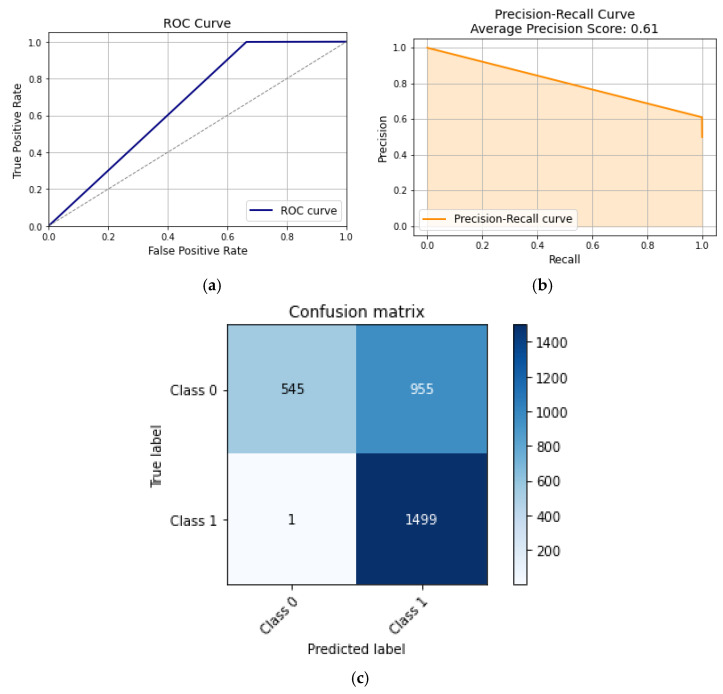
Random forest curves: (**a**) ROC curve, (**b**) precision–recall curve, and (**c**) confusion matrix.

Random forest is another classical machine learning algorithm that forms multiple decision trees independently. Taking the average or the most common outcomes for each decision tree forms the result of the random forest algorithm. Compared to decision trees, random forest had a slight but insignificant improvement compared to decision trees. These slight differences can be observed in Figure 10, where Figure 10a shows that the AUC is 67.5% compared to the decision trees, which had an AUC of 67.0%. Additionally, Figure 10b,c show that the precision–recall curves and the confusion matrix resemble that of decision trees, which indicates that using multiple decision trees for posture classification will have a negligible difference in accuracy and other metrics.

#### 5.2.5. SVM

**Figure 11:**
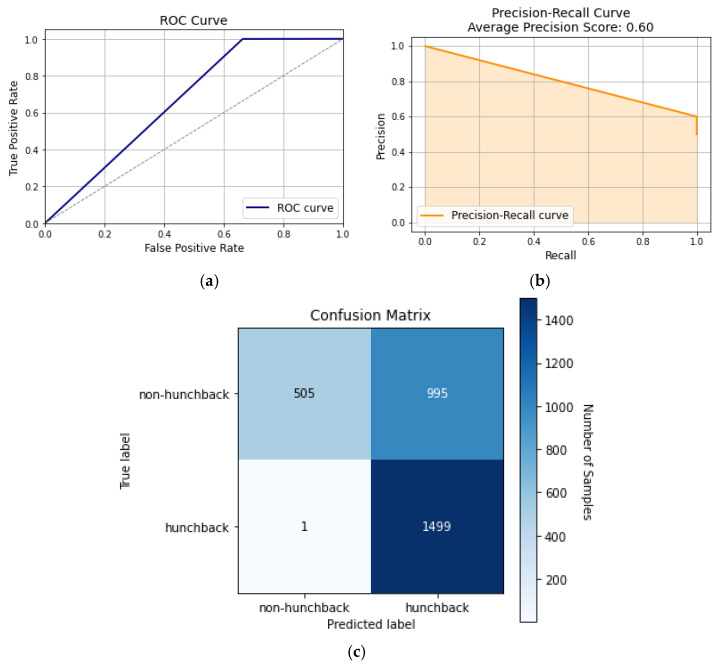
SVM curves: (**a**) ROC curve, (**b**) precision–recall curve, and (**c**) confusion matrix.

SVM is an ML algorithm that works well with high-dimensional datasets. SVM aims to find a hyperplane in N-dimensional space that can separate the different classes in the feature space. Even though the SVM can handle complex and non-linear data, it is susceptible to noise, preventing the model from generalizing the data to unseen data. Therefore, issues arise when applying the SVM to posture readings where the different features have a complex and dependent relation to one another. This can be observed in Figure 11, where the SVM did not produce high results in testing and had a similar performance to the decision tree and random forest algorithms.

Figure 11a shows that the AUC of the ROC curve is 67.0%, with average precision and macro-precision scores of 60% and 80%, respectively, as shown in Figure 11b. This indicates that the SVM has limited distinction between hunchback and non-hunchback positions while slightly leaning towards correct identification. Figure 11c shows the confusion matrix of the classification model, where it had TP and TN rates of 50% and 16.8%, while having an FP rate of 33.2%, which accounts for a third of all samples. Therefore, examining various neural networks and time-dependent models can help enhance accuracy and the metrics.

#### 5.2.6. MLP Classifier

The MLP classifier is a neural network model that is used for classification purposes in machine learning. The architecture of the MLP classifier involves an input layer, multiple hidden layers, with the number of layers being proportional to the complexity of the dataset, and an output layer. The complex relationship of the input parameters makes the MLP classifier a suitable model to use for hunchback classification. Tuning the hyperparameters, such as the activation function and the hidden layers’ size, optimizes the model’s performance. A common issue that some neural network models face is overfitting, which can be mitigated by applying regularization techniques such as dropout and weight decay. Compared to the other ML algorithms apart from KNN, the MLP classifier performs the best in accuracy, precision, recall, and F1 score.

**Figure 12:**
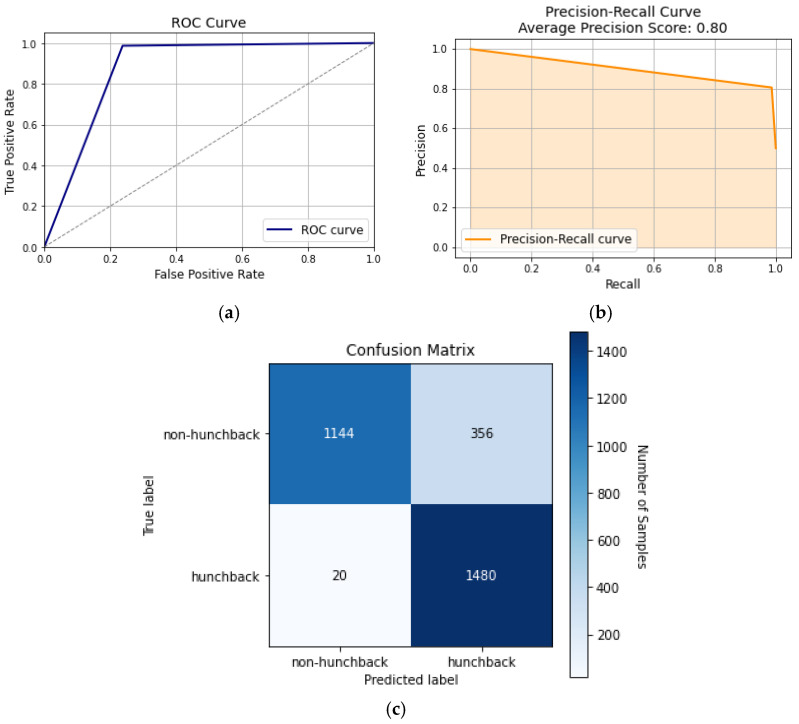
MLP classifier curves: (**a**) ROC curve, (**b**) precision–recall curve, and (**c**) confusion matrix.

Figure 12 displays the various curves for the classifier, which is shown to have significantly better results than some of the other models. In Figure 12a, the ROC has an area of 87%, meaning that it can distinguish between hunchback and non-hunchback positions, being surpassed only by KNN at 93%. Figure 12b displays the classifier’s precision–recall curve, which has an average precision score of 0.80, showing that the model has a good balance between precision and recall. This balance between precision and recall suggests that the model performs well in capturing and classifying positive instances. Figure 5c shows the confusion matrix of the MLP classifier, where most of the samples are correctly classified as TP and TN, giving average precision and recall rates of 89% and 87%, respectively. This makes the MLP classifier perform better than the ML classifiers, except for KNN.

#### 5.2.7. LSTM

Even though some of the aforementioned models and algorithms such as KNN and the MLP classifier can distinguish between hunchback and non-hunchback positions, these models are time-independent, which creates issues in capturing posture changes and bodily dynamic patterns. The LSTM model is a time-dependent and sequential recurrent neural network (RNN) that can capture temporal dependencies and sequence predictions. Since a hunchback posture and UCS vary over time, this makes LSTM a more suitable model to use for classification compared to the time-independent models.

**Figure 13:**
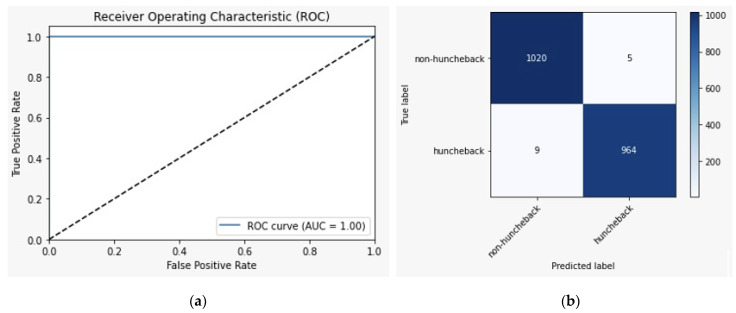
LSTM model curves: (**a**) ROC curve and (**b**) confusion matrix.

Alongside the enhanced accuracy rates, Figure 13a shows that the LSTM model has an AUC of more than 0.99. This AUC score indicates that the model performs well as it can always distinguish between hunchback and non-hunchback postures. Figure 13b displays the model’s confusion matrix, which has TP and TN rates of 48.2% and 51%, respectively. The high percentages of the TP and TN rates indicate that the model will predict hunchback and non-hunchback positions correctly with minimal error, since the FP and FN rates are both less than 1%.

### 5.3. Experimental Results

The experiment aimed to compare various models in posture classification, focusing on predicting hunchback occurrences. The findings revealed distinct performance variations among these models, with LSTM standing out as the top performer among all models. Notably, KNN demonstrated superior performance compared to the other time-independent models, although not reaching the accuracy levels of LSTM. Table 3 presents a comprehensive view of the models’ performance based on accuracy, AUC score, precision, recall, and F1 score. In particular, KNN’s effectiveness stemmed from its utilization of distance as a metric, contrasting with Naïve Bayes and decision trees, which treated individual features as independent. This approach enabled KNN to achieve a 92.9% accuracy in correctly classifying hunchback and non-hunchback postures, with the AUC score reflecting its high performance of 93%. This shows that KNN is able to correctly capture most of the positive instances of the classification, as reflected by the precision, recall, and F1 scores. Conversely, Naïve Bayes and decision trees, due to their assumption of feature independence, depicted poor accuracy, AUC, and F1 scores, with decision trees slightly outperforming Naïve Bayes but still exhibiting inadequate results. Naïve Bayes, in particular, suffered from a critical drawback by assuming conditional independence, leading to a mere 50% accuracy rate, essentially equating to random guessing. Employing an ensemble method like random forest, which aggregates the outcome of multiple decision trees, resulted in minor enhancements across accuracy, AUC score, precision, recall, and F1 score. However, the improvements were relatively minor. SVM presented poor results, similar to decision trees, with only precision having a slightly high result of 80%, indicating accurate predictions of positive instances. However, its recall of 67% reveals a tendency to miss some positive instances. A neural network model in the form of an MLP classifier showcased consistent performance across different metrics at around 87%, surpassing the other time-independent models, except KNN. Finally, LSTM, which is dependent on time, emerges as the most superior model, outperforming all the models, including KNN. With an accuracy, AUC score, precision, recall, and F1 score all exceeding 99%, LSTM highlighted the significant advantages of time-dependent models in classification tasks. The model’s classification capability enabled it to capture posture variations over time, making its performance remarkable.

Testing and experimenting with the different models give an overview over the stability, sensitivity, and specificity of the model. It also allows the response time to be obtained. Among the time-independent models, KNN stood out for its superior performance. It exhibited remarkable stability compared to the other models. While each training session captures slightly different data, it was able to perform well regardless of the data fluctuations among the trained models. Even though the inherent variations persisted, the KNN model was still able to distinguish between hunched and non-hunched postures. Its stability can be attributed to the nature of the KNN model, where it uses distance as a metric and is based on different interacting variables. Models like decision trees rely heavily on the original training data for predictions. In contrast, KNN’s non-parametric nature enabled it to dynamically adjust to data changes, consistently delivering reliable results in both the training and testing phases.

Other crucial metrics used to assess the performance of the model are the sensitivity and specificity, where the sensitivity measures the proportion of true positives among all the actual positives, i.e., the recall for the true positive class. Meanwhile, the specificity is the proportion of true negatives among all the actual negatives, i.e., the recall for the false positive class. These two metrics show how reliable the diagnostic and classification model is in capturing the true positive and negative. Table 4 shows the accuracy, sensitivity, and specificity for the KNN and LSTM models. By comparing the time-dependent and time-independent models, LSTM has higher accuracy, sensitivity, and specificity rates of 99.20%, 99.13%, and 99.51%, respectively. Furthermore, its sensitivity and specificity rates have less variations, meaning that the model captures both the positive and negative classes of hunchback and non-hunchback postures. On the other hand, KNN has slightly lower accuracy and sensitivity rates of 92.90% and 93.00% and a lower specificity rate of 86.20%. This means that the model has a preference in capturing the positive instances more than the negative instances. In terms of diagnostics, it is essential to balance the sensitivity and specificity with a high sensitivity, ensuring that individuals with hunchback postures will not be missed, while a high specificity ensures that people who are non-hunchback are less likely to be misclassified as hunchback. Through experimentation with these two models, it was demonstrated that LSTM outperformed KNN in correctly identifying and capturing individuals without hunchback. Even though both models had accuracy rates above 90%, the LSTM model was more accurate in classifying hunchback and non-hunchback postures among the tested subjects. The different metrics show the advantages that the time-dependent methods have against the time-independent methods, making them effective classification models.

**Figure 14:**
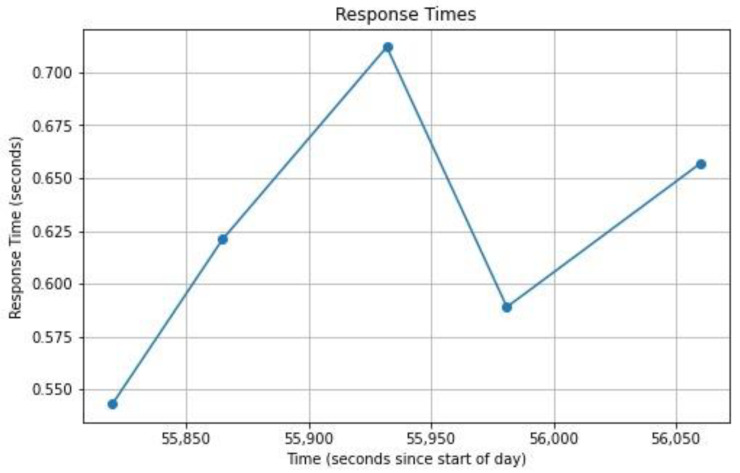
Response time at different requests.

Alongside the stability, repeatability, sensitivity, and specificity, the response time helps to show how responsive the model is when interacting with the two servers. Ideally, the response time should be as short as possible by minimizing delays and latencies. The system hinges on swift communication between the local Flask server and the Raspberry Pi. The goal is to maintain a response time under 1 s for real-time reading classification. An analysis of the response times recorded between these servers consistently showed durations between 0.543 and 0.712 s, as can be seen in Table 5 and Figure 14. The Figure shows a graphical representation of the different response times, as presented in Table 5. The range consistently meets our target threshold, with an average response time of 0.6244 s. There are multiple factors that create differences between each response time. These factors include a high number of load requests on the server, high latency between the servers, and the accumulation of requests in a queue waiting process. These factors cause slight variations between each response time. All in all, this swift exchange ensures the timely classification of real-time data. Meeting our response time goal signifies the efficiency of this communication, which is crucial for seamless data processing.

## 6. Conclusions and Future Work

The shift to a sedentary lifestyle has increased postural problems, such as hunchback, which is a vital sign of UCS. This paper aimed to detect and monitor UCS by correlating it to the hunchback posture. Given the prevalence of slouched and hunchback postures in society, particularly in the Middle East [2], the system can identify these issues in individuals. Identifying these issues will help alleviate the effects of UCS by correcting an individual’s posture and promoting healthier habits. As people’s well-being improves, productivity will increase, which can help individuals perform their jobs more consistently and for longer periods. This is particularly aligned with the UN sustainability goals, which aim to raise the standard of living and improve the overall quality of life. Therefore, having a back brace that detects and monitors postural issues will positively impact society. By enabling users to observe their progress over time, they can understand how a back brace corrects their posture and to what extent their posture is improving. Similarly, integrating doctors’ features into the mobile application allows them to evaluate the effectiveness of the treatment plan, saving time and costs associated with in-person visits. Overall, a smart corrective back brace facilitates the detection and monitoring of UCS, enabling patients to observe and improve their posture for long-term health benefits.

This system offers numerous advantages to both patients and healthcare providers. Its real-time progress allows patients to actively engage in their treatments, facilitating improved communication with doctors and streamlining the treatment process. Moreover, the system’s posture detection capability encourages individuals to seek timely medical attention when poor posture is detected. Its wireless functionality, reliant solely on local Wi-Fi for connectivity to the mobile application, enhances convenience and accessibility. However, a notable limitation is its inability to definitively diagnose UCS. Although it effectively identifies signs of UCS by detecting associated Hunchback posture, official medical diagnosis is still necessary to confirm UCS. Anticipated future advancements aim to enhance accuracy and directly detect UCS, enabling more precise diagnoses in patients.

This paper focused on detecting and monitoring signs of posture problems, specifically UCS, via certain postures such as hunchback. The back brace can be extended to include the detection of stress through back posture. This extension would aid in evaluating and displaying a user’s stress levels while using the brace. Additionally, one of the primary causes of UCS is FHP, which is linked to respiratory and cardiovascular illnesses. Therefore, a back brace can involve detecting FHP and identifying the various illnesses caused by it. The literature indicates additional avenues to explore, including stress, cardiovascular, and respiratory illnesses, all of which are detrimental to a person’s overall well-being. Different wearables, such as smart watches, can be used to alert the user if they are feeling stressed or anxious and give helpful tips on how to reduce stress. In addition to exploring different illnesses associated with FHP, the physical brace can be improved. This could be achieved by attaching additional sensors on external straps without inconveniencing the user. A neck strap can also be incorporated to have direct access to the nape of a person’s neck to identify FHP more easily. These additional sensors can help detect UCS and other underlying disorders more efficiently, such as turtleneck syndrome, which can help reduce societal postural problems. The examples above highlight the potential to innovate this field, creating highly functional and sophisticated systems that make healthcare more effective, efficient, and convenient.

## Figures and Tables

**Table 1:** Related works on wearable devices.

Wearable Technology	Sensor Location	Approach	Conclusion of the Study	Ref.
YEI 3-Space IMU Sensor	Back and safety helmet	The algorithm records the maximum duration for each real-time angle in every frame, which is then compared to the expected duration for operational postures.	The system was able to detect if the user was in a risky posture and send alerts.	[13]
Shimmer IMU	Cervical, thoracic, and lower lumbar spine	A signal filter was applied to all IMU data for feature extraction. The Symbolic Aggregate Approximation method was used for posture classification.	Successful classification and differentiation between hunchback and slouch back.	[14]
Zishi: 9-axis Adafruit IMU sensor	Spine and shoulder	Various statistical methods used for classification.	The vest can provide postural analysis and alert the user to correct their posture.	[15]
SPoMo: six-axis IMU (accelerometer and gyroscope)	Upper back and lower back	Utilized low-pass filtering and an explicit complementary filter for attitude estimation.	The system was able to monitor sitting spinal posture and if a user was sitting in a proper manner.	[16]
Smart garment: IMU sensors, metal composite embroidery yarn	IMU sensors: left and right shoulder, left and right waist	The IMU module data and camera data underwent filtering: a moving average filter was used in the last three data points, while a low-pass filter was applied to the camera data.	Accurate estimation of postural tilt of the torso.	[17]
A microcontroller and IMU sensor to capture movements	IMU sensors on the chest	The IMU data were classified using convolutional neural networks (CNNs) and long short-term memory (LSTM). These models predicted various activities such as walking, jogging, jumping, and sitting.	Accurate prediction of various activities.	[18]
Textile-based wearable using an inductive sensor embedded on a fabric	Inductive sensor sewed by a copper wire into a zigzag pattern	The wearable system identifies specific trunk movements such as bending, twisting, and lateral movements.	Lightweight, low power, and can accurately distinguish and detect trunk movements.	[19]
Wearable sensor in monitoring the lower back	Two sensors: the trunk IMU and pressure insoles under the feet	Monitored low back loading based on individuals performing manual intensive tasks. Used Gradient Boosted decision tree and an ensemble of 100 trees.	Provides an accurate and automated way to monitor lumbar movements to examine low back injury.	[20]
IMU sensors for gait analysis	The IMU sensor was placed at the lateral side of the leg	Used feedforward neural networks (FNNs) and LSTM to estimate nine activities.	The LSTM model had higher accuracy rates than FNN, showing its ability to enable gait analysis.	[21]
IMU sensors and pressure insoles	The IMU and pressure sensors were placed on the trunk and under the feet, respectively	Trunk signals were used as inputs for the machine learning model to estimate lumbar moment.	The pressure sensor helped accurately assess low back disorder.	[22]
IMU sensors	Three different locations: cervical vertebra, forehead, and occipital protuberance	Individuals performed exercise-based tasks which were compared to the standard.	The IMUs showed good reliability in the flexion and right lateral bending angles.	[23]
IMU embedded in wearable sensors	Upper limb of the individual	Calibration and measurement of IMUs to assess the quality in the upper limb.	Minimal change between the different input variables makes it possible to accurately assess upper limb movements.	[24]
Commercial depth camera	Camera and neckband used to capture the upper body	To detect FHP, they measured the distance between the torso and head using a depth camera, alongside assessing the degree of anteriority.	Accuracy rate of 98% with a false alarm rate of 2%.	[25]

**Table 2:** Detection of posture problems.

Posture Problem	System	Approach	Result	Ref.
Hunchback	Self-powered sitting position monitoring vest.	The signal is processed by a machine learning algorithm such as random forest, which produces accurate responses to different movements of the user.	Classify and detect 6 postures with 96.6% accuracy.	[32]
Neck posture	The system integrates a gyroscope, accelerometer, and magnetometer.	Noise was filtered using the moving average. The signals were processed per second and the action state was calculated as the median for each time interval.	Angle measurement error is less than 2%.	[27]
Slouched and hunchback postures	The system uses e-textile sensors and inertial sensors using resistive stretchable fabric.	A signal filter was applied to all IMU data for feature extraction. After extracting their desired features, they used the Symbolic Aggregate Approximation method for posture classification.	The classification model had an accuracy of 85% and was able to distinguish hunchback and slouched postures.	[14]
Postural deformities such as hunchback and slouched postures	Three accelerometers were placed on the neck, shoulder, and back to identify postural deformities.	Machine learning approaches were utilized for classification, which includes Support Vector Machine (SVM) and isolation forest classification.	Identify postural problems with accuracy rates up to 99.3%.	[33]
Spin postures in different directions and deformities	The system consists of a shirt with built-in magnets that work with a magnetic sensor placed above the body’s sternum.	The sensor recorded data at 25 Hz, with the data passing through a lowpass Butterworth filter to eliminate unwanted noise. The resulting data were then processed in MATLAB.	The system can detect lower body postures and changes in crossed legs.	[34]
Four pose classes: Correct Pose, Incorrect Neck Pose, Incorrect Shoulder Balance, and Incorrect Arm Elevation Pose	The system consists of a belt that has an IMU, three LEDs, and an HD camera.	The IMUs collect the thoracic and thoracolumbar angles and the HD camera recorded the instances of the user. The result is transmitted via a cloud server where a machine learning algorithm is trained to view and track seating posture overtime.	The model was able to detect correct poses with 96% accuracy.	[35]

**Table 3:** Different classifier metrics.

Classifier	Accuracy	AUC Score	Macro-Avg. Precision Score	Macro-Avg. Recall Score	Macro-Avg. F1 Score
Decision trees	66.50%	67.00%	80.00%	67.00%	62.00%
Naïve Bayes	50.00%	50.00%	25.00%	50.00%	33.00%
KNN	92.90%	93.00%	94.00%	93.00%	93.00%
Random forest	68.13%	67.50%	80.00%	68.00%	65.00%
SVM	66.80%	67.00%	80.00%	67.00%	63.00%
MLP classifier	87.47%	87.00%	89.00%	87.00%	87.00%
LSTM (time-dependent)	99.20%	>99.00%	99.51%	99.13%	99.32%

**Table 4:** Sensitivity and specificity of different models.

Classifier	Accuracy	Sensitivity	Specificity
KNN	92.90%	93.00%	86.20%
LSTM	99.20%	99.13%	99.51%

**Table 5:** Response time testing at different requests.

Real Time	Response Time (s)
15:30:20	0.543
15:31:05	0.621
15:32:12	0.712
15:33:01	0.589
15:34:20	0.657

## Data Availability

Data are contained within the article.
